# Epidemiologic investigation on neurological complications following neuraxial anesthesia in 2.7 million cases in Southwest China

**DOI:** 10.1038/s41598-025-98142-5

**Published:** 2025-04-18

**Authors:** Li-pei Shu, Jie-mei Ji, Zhong-xuan Mao, Xiang-li Xiao, Jian Lai, Yu Dai, Shan-shan Wei, Kai-yuan Liang, Yang Zhao, Ya-jun He, Yu-nan Lin, Jing-chen Liu

**Affiliations:** 1 Department of Anesthesiology, The First Affiliated Hospital of Guangxi Medical University, 22 Shuangyong Road, Nanning, 530021 Guangxi China; 2 Department of Anesthesiology, People’s Hospital of Beihai City, Beihai, Guangxi China; 3 Department of Anesthesiology, Third People’s Hospital of Hechi City, Hechi, Guangxi China; 4 Department of Anesthesiology, People’s Hospital of Guigang City, Guigang, Guangxi China; 5 Department of Anesthesiology, Affiliated Hospital of North Sichuan Medical College, Nanchong, Sichuan China

**Keywords:** Combined spinal-epidural anesthesia, Epidural anesthesia, Epidemiologic investigation, Neurological complications, Spinal anesthesia, Epidemiology, Neurological disorders, Risk factors

## Abstract

Neuraxial anesthesia provides effective anesthesia and analgesia for surgery, but may cause neurological complications. The rate of neurological complications in China remains unclear. This study conducted a retrospective epidemiological investigation of neurological complications following neuraxial anesthesia in Guangxi, southwest China. This survey used the “Golden Data” platform to distribute questionnaires to anesthesiology departments across hospitals of varying levels in Guangxi, gathering data on neuraxial anesthesia methods and associated neurological complications from 2013 to 2022. Detailed patient information was recorded, with missing data supplemented by phone. The Adverse Event Reporting System was also utilized to verify and supplement cases, supported by peer review. The study analyzed the incidence and clinical characteristics of neurological complications after neuraxial anesthesia. A comprehensive survey was conducted across 243 hospitals, encompassing 2,723,615 cases of neuraxial anesthesia. The survey identified 1208 cases of neurological complications, with an incidence of 0.44‰, primarily occurring in patients undergoing obstetrics, gynecology, orthopedics, urology, and general surgery. The complications included transient nerve syndrome (999 cases), spinal injury (188 cases), cauda equina syndrome (13 cases), spinal hematoma (5 cases), anterior spinal artery syndrome (2 cases), and conus medullaris injury (1 case). The incidence of neurological complications associated with combined spinal-epidural anesthesia (0.53‰) was significantly higher than that of epidural anesthesia (0.21‰) and spinal anesthesia (0.35‰) (*P* < 0.001). Among the patients, 97.3% achieved full recovery, while 2.7% (30 cases) suffered permanent neurological damage. Although neurological complications are relatively rare, comprehensive preoperative assessment, adherence to standardized protocols, and vigilance regarding potential complications are essential.

## Introduction

Neuraxial anesthesia remains an important method in anesthesia practice due to its numerous advantages, including minimal equipment requirement, simplicity of drug administration, lower cardiovascular effects, expedited postoperative recovery, and applications in perioperative medicine for both postoperative analgesia and labor analgesia^1–5^. This technique is particularly prevalent in grassroots hospitals where access to general anesthesia may be limited. However, it is important to note that neurological complications can occur, potentially arising from spinal injury during the procedure or neurotoxicity of local anesthetics^6–9^. Numerous cases of neurological complication following neuraxial anesthesia have been reported^10–14^.

Currently, the majority of large retrospective or observational studies on the incidence of complications associated with neuraxial anesthesia are derived from closed malpractice claims, postal surveys, and literature reviews^15–19^. Furthermore, there is a paucity of studies that specifically address neurological complications resulting from neuraxial anesthesia. The study conducted by Brull et al. serves as a review of 32 studies involving neurological complications subsequent to neuraxial anesthesia and peripheral nerve blocks, revealing an incidence rate of neurological complications following neuraxial anesthesia of 0.4‰ in 2007^17^. A retrospective survey conducted in Japan examined neurological complications associated with neuraxial anesthesia, reporting an incidence of spinal cord injury of 0.013‰ among 548,819 cases of neuraxial anesthesia in 2007^20^. In another study, the incidence of severe neurological complications following central neuraxial blockades in Sweden was 0.074‰ among 1,710,000 blockades in 2004^18^. However, the incidence of neurological complications following neuraxial anesthesia in China remains unclear.

The primary categories of neurological complications include transient neurological syndrome (TNS), cauda equina syndrome (CES), spinal nerve root injury, anterior or posterior spinal artery syndrome, and neurological manifestations attributable to spinal hematoma or infection^21^. The extent of recovery is contingent upon the specific type of complication: patients with TNS generally attain full recovery, whereas other complications may result in enduring nerve damage. Such outcomes impose considerable psychological and economic burdens on patients, and may precipitate medical disputes. Therefore, it is imperative to critically collect and scrutinize data regarding neurological complications associated with neuraxial anesthesia. This study aimed to perform a retrospective epidemiologic investigation on the incidence and outcomes of neurological complications across 243 hospitals in the Guangxi Zhuang Autonomous Region, China, from 2013 to 2022.

## Materials and methods

### Survey

The survey, comprising a Microsoft Excel table and questionnaire, was disseminated via the “Golden Data” network platform to the heads of departments and clinical anesthesia quality controllers across all primary, secondary and tertiary hospitals of anesthesia departments in the Guangxi Zhuang Autonomous Region of China. This retrospective investigation received approval from the Ethics Committee of the First Affiliated Hospital of Guangxi Medical University (Approval No.: 2022-K064-01) and was registered with the Chinese Clinical Trial Registry (www.chictr.org.cn) under registration number ChiCTR2200065239 on November 1, 2022. The study was conducted in accordance with the ethical principles outlined in the Declaration of Helsinki. For all patients with neurological complications following neuraxial anesthesia that were surveyed, we obtained written informed consent from the patients or their guardians via phone.

The study recipients were instructed to document cases involving all anesthesia methods employed within their departments from 2013 to 2022, including epidural anesthesia (EA), combined spinal-epidural anesthesia (CSEA), spinal anesthesia (SA), general anesthesia (GA), peripheral nerve block (PNB), and other anesthesia techniques on the provided Excel sheet, as well as the number of neurological complications associated with neuraxial anesthesia. For departments documenting neurological complications, recipients were mandated to complete an additional questionnaire. The purpose of this questionnaire was to correlate each previously reported complication with specific patients. The data collected included the patient’s sex, age, surgical department, type of anesthesia administered, history of neuraxial anesthesia, use of postoperative epidural analgesia pumps, number of spinal or epidural punctures, local anesthetic concentration and dosage, any special events occurring during anesthesia, radiographic evaluations (including lumbar MRI or CT), and treatment and recovery outcomes with first year post-surgery. Questionnaires with incomplete information were obtained via telephone consultations. The risk of bias was not evaluated, because the study exclusively involved patients with neurological complications and only comprised clinical data and consequences.

### Adverse event reporting system

Parallel to the online investigation, adverse event reports pertaining to neurological complications following neuraxial anesthesia were examined using the direct reporting system of the medical adverse event network within the Guangxi Anesthesiology Department. To enhance the quality of clinical anesthesia in Guangxi, all departments, especially quality control specialists, are obligated to report anesthesia-related adverse events using this online system. Subsequently, all reported cases underwent peer review. We reviewed and scrutinized all instances of neurological complications associated with neuraxial anesthesia.

### Definitions

EA was defined as the insertion of an epidural catheter into the vertebral space via an epidural puncture needle, followed by the administration of a local anesthetic through the catheter. SA entails the injection of a local anesthetic using a thin spinal needle, which may be performed independently or facilitated by an epidural needle. Moreover, once spinal anesthesia was successfully administered during neuraxial anesthesia, the insertion of an epidural catheter, irrespective of whether the local anesthetic was administered intraoperatively or solely for postoperative epidural analgesia, was classified as CESA. Additionally, CESA was defined in cases where it was initially planned, but due to the absence of cerebrospinal fluid return from the spinal anesthesia needle, the decision was made to forgo subarachnoid anesthesia and proceed with the placement of an epidural catheter for the administration of EA.

Intravertebral hematomas can be situated within epidural or subdural spaces. In this study, all instances of intravertebral hematomas were referred to as “spinal hematomas”. This study exclusively considered cases of CES attributed to local anesthetic, either due to its toxic effects or presumed compression resulting from the volume effect of the local anesthetic. Patients with CES caused by spinal hematoma or epidural abscess were excluded from the CES group.

TNS was defined as the clinical evaluation and neurological examination of patients conducted by anesthesiologists. In cases of suspected or confirmed complications of other types, a neurological examination of the lower extremities by a neurologist within 24 h was mandated to collaboratively determine the diagnosis and treatment measures. Consequently, patients exhibiting confirmed or suspected neurological symptoms were included in the study on neurological complications following neuraxial anesthesia. Patients with pre-existing neurological symptoms or neuropathy prior to surgery were excluded from the study.

Spinal cord injuries can vary in severity, ranging from mild to severe. The clinical manifestations of mild spinal cord injury and spinal nerve root injury exhibit considerable overlap, often presenting as unilateral or bilateral paresthesia or numbness in the lower extremities or localized areas following neuraxial anesthesia, with or without accompanying muscle strength impairment. Consequently, distinguishing the specific type of neurological complication based solely on clinical symptoms and ancillary examinations may be challenging. For the purposes of this article, both spinal cord injury and spinal nerve root injury are collectively referred to as “spinal injury”. Permanent neurological injury was defined as symptoms persisting for more than one year.

## Results

### Number of anesthesia cases

Utilizing the survey compiled in Microsoft Excel, we gathered 9,555,023 cases of anesthesia in operating rooms across 243 hospitals from 2013 to 2022. Among these cases, neuraxial anesthesia accounted for 2,459,805 instances, representing 25.74% of the total, thereby constituting the largest proportion of anesthesia methods followed by general anesthesia.

### Neurological complications following neuraxial anesthesia

China initiated the promotion of labor analgesia in November 2018, prompting the commencement of data collection on labor analgesia cases from 2019 onwards. Between 2019 and 2022, a cumulative total of 263,810 instances of neuraxial analgesia for labor analgesia were recorded in Guangxi. Specifically, the annual figures are as follows: 75,643 cases in 2019, 70,527 cases in 2020, 53,671 cases in 2021, and 63,969 cases in 2022. The data on neurological complications encompassed labor analgesia following neuraxial anesthesia, resulting in an overall total of 2,723,615 neuraxial anesthesia cases.

The types of neurological complications following neuraxial anesthesia were TNS, spinal injury, CES, spinal hematoma, anterior spinal artery syndrome and conus spinal injury in this study. Neurological complications following neuraxial anesthesia were documented across 162 hospitals, resulting in a total of 1144 cases, as indicated in the Microsoft Excel dataset. An additional 64 cases were identified solely through the direct reporting system for adverse events, which included 44 instances of TNS, 15 cases of spinal injury, three occurrences of CES, and two instances of spinal hematoma. All cases retrieved from the direct reporting system were analyzed by members of the quality control team, who determined that these complications were attributable to neuraxial anesthesia. Detailed clinical case records were obtained for each instance. The total number of neurological complications was 1208 (see Table 1). In addition to these neurological complications, several other complications associated with neuraxial anesthesia have been reported in the adverse event reporting system. These included residual epidural catheter fracture, high spinal anesthesia, total spinal anesthesia, excessively wide epidural block levels, headache following epidural rupture, and central toxicity of local anesthetics. However, they were excluded from the analysis because they were not classified as neurological complications.

**Table 1:** Neurological complications and information sources (n).

Types of complications	Number of cases in survey	Number of cases in adverse event reporting system	Total	Incidence (%)
TNS	955	44	999	82.7
Spinal injury	173	15	188	15.5
CES	10	3	13	1.1
Spinal hematoma	3	2	5	0.4
Anterior spinal artery syndrome	2	0	2	0.2
Conus spinal injury	1	0	1	0.1
Total	1144	64	1208	100

*TNS* Transient nerve syndrome; *CES* Cauda equina syndrome.

Among the various types of neurological complications, TNS constituted the majority (82.7%), followed by spinal injury (15.5%), CES (1.1%), and a combined incidence of 0.7% for spinal hematoma, anterior spinal artery syndrome, and conus medullaris injury (see Table 1).

Between 2013 and 2022, a total of 2,723,615 cases of neuraxial anesthesia were recorded, along with 1208 instances of associated neurological complications, resulting in a neurological complication incidence rate of 0.444‰. Notably, 818 (67.7%) of these complications occurred between 2013 and 2017. The incidence of complications has exhibited a downward trend from 2013 to 2022. A greater incidence of neurological complications was observed following CSEA, with a rate of 0.53‰, which was significantly higher than the rates associated with EA at 0.21‰ and SA at 0.35‰ over the 10-year survey period. Furthermore, the incidence of neurological complications following SA was higher than that associated with EA (see Table 2).

**Table 2:** Neurological complications following neuraxial anesthesia from 2013 to 2022 (n/‰).

	EA			CSEA			SA			Neuraxial anesthesia		
	NC	Cases	INC	NC	Cases	INC	NC	Cases	INC	NC	Cases	INC
2013	36	48,154	0.748	149	143,337	1.040	5	6265	0.798	190	197,756	**0.961**
2014	26	44,963	0.578	144	158,096	0.911	4	7387	0.541	174	210,446	**0.827**
2015	19	44,274	0.429	129	159,360	0.809	3	7820	0.384	151	211,454	**0.714**
2016	14	42,983	0.326	138	167,858	0.822	8	8317	0.962	160	219,158	**0.730**
2017	15	40,981	0.366	119	181,705	0.655	9	11,175	0.805	143	233,861	**0.611**
2018	7	48,037	0.146	53	224,311	0.236	1	20,074	0.050	61	292,422	**0.209**
2019	16	127,345	0.126	52	233,065	0.223	4	19,426	0.206	72	379,836	**0.190**
2020	6	117,978	0.051	60	227,703	0.264	2	13,503	0.148	68	359,184	**0.189**
2021	6	83,311	0.072	69	204,135	0.338	5	21,862	0.229	80	309,308	**0.259**
2022	8	93,124	0.086	94	195,713	0.480	7	21,353	0.328	109	310,190	**0.351**
合计	**153**	**691,150**	**0.211**^ab^	**1007**	**1,895,283**	**0.531**	**48**	**137,182**	**0.350**^a^	**1208**	**2,723,615**	**0.444**

Compare with CSEA, ^a^*p* < 0.001. Compared with SA, ^b^*p* < 0.001. NC, number of cases with neurological complications; Cases, number of cases with related anesthesia methods; INC, the incidence rate of neurological complications; *EA* Epidural anesthesia; *CSEA* Combined spinal and epidural anesthesia; *SA* Single spinal anesthesia.

Significant values are in bold

### Neurological complications in different anesthesia methods and surgical types

Among the three types of neuraxial anesthesia, neurological complications following CSEA constituted the majority, accounting for 83.6% of cases. Notably, of the 13 cases of CES, 12 cases were associated with CSEA (see Table 3).

**Table 3:** Neurological complications by types of neuraxial anesthesia and surgery (n).

	Neuraxial anesthesia methods			Surgery types				Total
	EA	CSEA	SA	Gynaecology and Obstetrics	Orthopedic surgery	Urology	General surgery	
TNS	116	852	31	593	208	95	103	999
Spinal injury	33	139	16	127	32	8	21	188
CES	1	12	–	11	1	1	–	13
Spinal hematoma	3	2	–	–	–	4	1	5
Anterior spinal artery syndrome	–	2	–	1	–	–	1	2
Conus spinal injury	–	–	1	1	–	–	–	1
Total	153	1007	48	733	241	108	126	1208

*EA* Epidural anesthesia; *CSEA* Combined spinal and epidural anesthesia; *SA* Single spinal anesthesia; *TNS* Transient nerve syndrome; *CES* Cauda equina syndrome.

Given that gynecology and obstetrics are often housed within the same departmental framework in numerous hospitals, this study analyzed them collectively. Notably, neurological complications predominantly occurred in obstetric and gynecological populations, constituting 60.7% of all complications. This was followed by orthopedic surgery at 20.0%, general surgery at 10.4%, and urological surgery at 8.9% (see Table 3).

### Clinical information of patients with neurological complications

Table 4 presents a summary of clinical data of the 1,208 patients who experienced neurological complications following neuraxial anesthesia. Notably, the proportion of female patients exceeded that of male patients, which may be attributed to the greater number of obstetric and gynecological surgeries than general surgery or orthopedic procedures. Additionally, 253 patients (20.9%) had a documented history of neuraxial anesthesia.

**Table 4:** Clinical data for 1208 patients with neurological complications following neuraxial anesthesia.

		EA	SA	CESA	Total
Sex (n)	Male	69	27	302	**398**
	Female	84	21	705	**810**
Age (year)		47.6 (25.5, 77.8)	55.5 (19.7, 86.9)	34.9 (28.4, 59.1)	
Surgery type (n)	General surgery	24	16	86	**126**
	Orthopaedic surgery	19	21	201	**241**
	Gynaecology and Obstetrics	82	8	643	**733**
	Urology	28	3	77	**108**
History of neuraxial anesthesia (n)	Yes	33	9	211	**253**
	No	120	39	796	**955**
Anesthesiologist Title (n)	Junior resident physician	46	6	243	**295**
	Senior resident physicians and junior attending physicians	75	27	459	**561**
	Senior attending physicians and associate Chief/Chief physicians	32	15	305	**352**
Anesthesia time (mins)		182.2 ± 53.3	66.7 ± 17.1	146.3 ± 38.2	
Number of punctures needle (n)	1	104	19	587	**710**
	2	17	7	71	**94**
	≥ 3	7	2	48	**57**
Puncture space (n)	L3-L4	21	43	902	**966**
	L2-L3	48	5	105	**158**
	L1-L2	58	0	0	**58**
	higher than L1	26	0	0	**26**
Depth of epidural catheter placement (n)	3–4 (≤ 4) cm	100	0	676	**776**
	4–5(≤ 5) cm	51	0	325	**376**
	≥ 6 cm	2	0	6	**8**
Local anesthetic type and concentration	Ropivacaine	0.375–1%	0.5–0.75%	0.5–0.75%	
	Bupivacaine	0.375–0.5%	0.25–0.75%	0.25–0.75%	
	Lidocaine	0.5–2%	–	–	
Abnormal sensation during puncture (n)	Yes	24	6	167	**197**
	No	109	42	571	**722**
Epidural analgesia pump (n)	Yes	81	0	485	**566**
	No	72	0	522	**594**

*EA* Epidural anesthesia; *CSEA* Combined spinal and epidural anesthesia; *SA* Single spinal anesthesia.

Significant values are in bold

In the recorded for the number of neuraxial punctures, 710 cases (82.5%) were successful on the first attempt, while 57 cases (6.6%) necessitated three or more “in and out” needle passes during the procedure. The primary reasons for multiple punctures included unclear puncture sites, catheter dislodgment, patient non-compliance, and requirement for re-puncture due to patients experiencing an abnormal sensation during the procedure. Among the 1208 cases of neurological complications, 347 cases lacked documentation regarding the number of epidural or lumbar needle punctures and 289 cases did not record abnormal sensory information. In total, 197 patients (21.4%) reported experiencing abnormal sensations during the puncture process.

Among patients who underwent SA and CESA, 945 (89.6%) underwent puncture at the L3-L4 interspace, whereas 110 (10.4%) went puncture at the L1-L2 interspace. After surgery, 566 patients (48.8%) used an epidural patient-controlled analgesia pump for 2–3 days; data on analgesia pump usage were not recorded for 48 cases.

Of the 1208 patients with neurological complications, only 393 had available lumbar CT or MRI data. Among these, the imaging results of 104 patients revealed no significant abnormalities. Moreover, 289 patients exhibited various pathological findings, including disc herniation (degeneration, protrusion, posterior protrusion) at different lumbar segments, interspinous ligament edema, Schmorl’s node formation at vertebral edges, nerve root compression, bilateral lateral recess narrowing, and lumbar vertebrae osteophytes.

### Recovery of neurological complications

Of the 1144 questionnaires received, 75 patients with TNS and 19 patients with spinal injury were recorded only numerically and anesthesia methods, without specific information and patient outcomes. One patient with CES had severe symptoms and was transferred to a superior hospital for treatment, which was considered incomplete recovery and permanent neurological injury. All 64 complications were reported in detail using the adverse event recording system. Therefore, a total of 1114 patients with neurological complications had specific information, 1050 from the questionnaires and 64 from the direct reporting system of adverse event. The overall recovery rate for neurological complications was 97.3%, with 30 cases resulting in permanent neurological injury (see Table 5).

**Table 5:** Recovery status of patients with neurological complications (n).

Complications	Full recovery	Permanent neurological injury	No information	All	Recovery rate (%)
TNS	924	0	75	999	100
Spinal injury	155	14	19	188	91.7
CES	4	9	–	13	30.8
Spinal hematoma	1	4	–	5	20.0
Anterior spinal artery syndrome	0	2	–	2	0.0
Conus spinal injury	0	1	–	1	0.0
Total	1084	30	94	1208	97.3

*TNS* Transient nerve syndrome; *CES* Cauda equina syndrome.

Permanent nerve injury could be minor or major. Among the patients with spinal injury, 155 (91.7%) achieved full recovery, while 14 experienced permanent neurological damage, with detailed clinical information available in Table 6. Of these 14 cases, 12 involved minor injury and two were major injury. Details information regarding patients with other serious neurological complications, such as CES, epidural hematoma, anterior spinal artery syndrome, and conus spinal injury, is provided in Table 7.

**Table 6:** Clinical information on patients with spinal injury causing serious consequences.

Case number	Gender (F/M), age(yrs)	Past history and comorbidities	Surgery type	Anesthesia	Comments anesthesia/analgesia	Symptoms	Radiography	Recovery result	Medical appraisal and compensation
1	F,56	hypertension	appendectomy	CSEA, L2-3	Electric sensation during puncture	Numbness and weakness of right calf	CT: no abnormal	PMII	Yes
2	F,41	Neuraxial anesthesia	internal fixation of thoracic vertevral fracture with nail rod	CEA, T12-L1	Two puncture, electric sensation during puncture	Numbness and weakness of left lower extremity	NA	PMII	Yes
3	M,45	No	Debridement of left anterior malleolus and flap transplantation	CSEA, L2-3	Electric sensation during puncture, epidural analgesia	Numbness of the right extremity, inability to walk	MRI: L4-L5 disc herniation	PMAI, right lower extremity hypoesthesia and muscle atrophy, right foot dorsiflexion weakness	Yes
4	M,36	No	Cesarean delivery	CSEA, L3-4	Uneventful puncture, epidural analgesia	Left calf flexor weakness with skin numbness	NA	PMII	No
5	M,27	No	Cesarean delivery	CSEA, L3-4	Difficult puncture with three spinal attempts (L3-4, L2-3 and L1-2 lastly), electric sensation during puncture	Left foot skin numbness and weakness	NA	PMII	Yes
6	M,62	Lumbar trauma	Ovariectomy	CSEA, L2-3	Uneventful puncture, epidural analgesia	Needle-like pain in left ankle joint	MRI: lumbar hyperosteogeny, L2-L5 disc herniation	PMII	Yes but no compensation
7	M,28	No	Cesarean delivery	CSEA, L2-3	Electric sensation during puncture, epidural analgesia	Pain in the right hip and lower extremity	CT: mild lumbar disc herniation	PMII	Yes
8	M,37	No	Cesarean delivery	CSEA, L2-3	Difficult puncture with three spinal attempts, electric sensation during puncture	One side of the lower extremity weakness, foot numbness	NA	PMII	No
9	F,40	No	Bone marrow harvesting	CSEA, L3-4	Difficult puncture with three spinal attempts, electric sensation during puncture	Right lower extremity pain, numbness and weakness, limited toes movement	MRI: Mild L4-L5 disc herniation and spinal stenosis, Ankylosing spondylitis?	PMII	No
10	M,37	hypertension	Cesarean delivery	CSEA, L3-4	Electric sensation during puncture, epidural analgesia	No dorsiflexion of the right foot, numbness of right lower extremity	MRI: lumbosacral fasciitis at l2-L3 level	PMII	No
11	M,56	No	Ureteroscopic lithotripsy	CSEA, L2-3	Difficult puncture with three spinal attempts, electric sensation during puncture	Pain in left thigh and weakness in left lower extremity	MRI: abnormal signal in the lumbar spinal	PMII	Yes
12	M,52	No	Removal of left tibial plateau with internal fixation	CSEA, L3-4	two puncture, electric sensation during puncture	Numbness in the right buttocks, outer thigh and plantar, waist pain and limited movement	MRI: L4/5, L5/S1 disc herniation, L4/5 interspinous ligamentitis	PMII	Not recorded
13	M,55	No	Ureteroscopy	CSEA, L3-4	electric sensation during puncture	Lower back and right lower extremity pain, right lower limb weakness	NA	PMII	Not recorded
14	M, 64	No	Appendectomy	SA, L2-3	Lumbar kyphosis with five punctures, electric sensation during puncture	Weakness and numbness in the right lower limb	Spinal cord injury	PMAI, weakness and numbness in the right lower limb	Yes

*EA* Epidural anesthesia; *CSEA* Combined spinal and epidural anesthesia; *SA* Single spinal anesthesia; *PMII* Permanent neurological minor injury; *PMAI* Permanent neurological major injury.

**Table 7:** Clinical information on patients with other serious neurological complications.

Case number	Gender(F/M), age (yrs)	Past history and comorbidities	Surgery type	Anesthesia	Comments anesthesia/analgesia	Symptoms	Complication	Radiography	Recovery result	Medical appraisal and compensation
1	M,30	Neuraxial anesthesia	Cesarean delivery	SA, L2-3	Uneventful puncture	Numbness and pain in both lower extremity	Conus spinal injury	MRI: T12-L1 spinal cord injury	PMAI: left foot weakness and difficulty walk	Yes
2	M,28	Neuraxial anesthesia	Cesarean delivery	CSEA, L3-4	Uneventful puncture, epidural analgesia	weakness in left lower extremity	Anterior spinal artery syndrome	MRI: L5/S1 disc degeneration and herniation	PMII	Yes but no compensation
3	M,47	No	appendectomy	CSEA, L2-3	Uneventful puncture, epidural analgesia	No sensation in left foot, unable to walk	Anterior spinal artery syndrome	MRI: L3/4, L4/5, L5/S1 disc herniation and spinal demyelination	PMAI: numbness and weakness in left lower extremity, can’t walk	Yes
4	M,75	Hypertension	Exploratory laparotomy	CEA, T9-10	Uneventful puncture, epidural analgesia	Lumber back pain, limb weakness	Epidural hematoma	MRI: T2-T8 extramedullary occupation: epidural hematoma with spinal cord compression and edema	PMII: operative treatment	No
5	F,32	Ankylosing spondylitis	Double J tube placement for renal calculi	CSEA, L3-4	Difficult puncture with five spinal attempts, electric sensation during puncture, epidural analgesia	High paraplegia, endotracheal intubation	Epidural hematoma	MRI: C2-L1 epidural hematoma	PMAI	No
6	F, 74	No	Prostate tumor biopsy	CSEA, L2-3	Uneventful puncture	Pain in lower back and left lower extremity, decreased muscle strength in the left lower extremity, hypoesthesia of the right lower extremity	Epidural hematoma	MRI: Abnormal signal shadow behind the spinal canal of T10-L3, epidural hematoma suspected; Herniated discs in T8/9 to L5/S1	After conservative: PMII	No
7	F, 68	No	TURP	CEA, L2-3	Difficult puncture twice	Pain in the lumbosacral region is excruciating	Epidural hematoma		After operation: recover	No
8	F, 64	No	Ureteroscopic lithotripsy	CSEA, L2-3	Uneventful puncture	Hypoesthesia and motor deficits of lower limb, Incontinence	Epidural hematoma	epidural hematoma, about 6mm*5mm in size	After conservative: PMII	Yes but no compensation
9	M,32	No	6 h labor analgesia followed by a cesarean delivery	CSEA, L3-4	Uneventful puncture, epidural analgesia	Urinary incontinence, numbness and weakness in left lower extremity	Cauda equina syndrome	CT: no abnormal	Recover	Yes but no compensation
10	M,31	No	Cesarean delivery	CSEA, L2-3	electric sensation during puncture, epidural analgesia	weakness in left lower extremity and unable to walk	Cauda equina syndrome	NA	PMII	No
11	M,32	No	Cesarean delivery	CSEA, L3-4	Uneventful puncture, epidural analgesia	Perianal numbness, difficulty urinating and defecating	Cauda equina syndrome	MRI: no abnormal	PMII	Yes
12	M,64	No	Lower extremity internal fixation removal operation	CEA, L2-3, attempt	Difficult puncture three times, CEA failed, instead of GA	Weakness and sensory disturbance in both lower extremity	Cauda equina syndrome	NA	Recover	No
13	M,28	No	Cesarean delivery	CSEA, L3-4	Uneventful puncture, epidural analgesia	Incontinent of urine and feces, no sensation and strength in right lower extremity, numbness and weakness in left	Cauda equina syndrome	MRI: no abnormal	PMAI: unable to walk, still incontinent	Yes
14	M,42	Neuraxial anesthesia	Cesarean delivery	CSEA, L2-3	Uneventful puncture, epidural analgesia	Weakness in both lower extremity	Cauda equina syndrome	CT: lumbar hyperosteogeny	Recover	No
15	M,38	Diabetes	4.5 h labor analgesia followed by cesarean delivery	CSEA, L3-4	Uneventful puncture, epidural analgesia	Incontinence of urine and feces, numbness of lower limbs (and weakness), buttocks and perineum	Cauda equina syndrome	MRI: no abnormal	PMAI: numb hips, lack of perineal feeling, weak bladder and bowel control	No
16	M,38	No	Cesarean delivery	CSEA, L3-4	Uneventful puncture, epidural analgesia	No sensation and strength in lower extremities	Cauda equina syndrome	MRI: no abnormal	PMAI: numbness and weakness in lower extremities	Yes
17	M,30	No	Cesarean delivery	CSEA, L3-4	electric sensation during puncture, epidural analgesia	Incontinence of urine and feces, paralysis	Cauda equina syndrome	CT: L4-5, L5-S1 disc herniation, MRI: suggested no spinal injury, possible injury to the left sciatic nerve	PMAI: normal muscle strength of lower extremities, weak control of urine and feces	No
18	M, 28	No	Cesarean delivery	CSEA, L2-3	Uneventful puncture, epidural analgesia	Weakness in both lower extremity	Cauda equina syndrome	MRI: no abnormal	PMII	Yes but no compensation
19	M, 26	Pregnancy-induced hypertension syndrome	Cesarean delivery	CSEA, L3-4	Patient obesity, Epidural puncture three times, epidural analgesia	Intestinal obstruction, urinary retention, numbness in both lower limbs	Cauda equina syndrome	MRI: no abnormal	Recover	No
20		No	Open reduction and internal fixation of femoral fracture	CSEA, L3-4	Uneventful puncture, epidural analgesia	Numbness in perineal, dysuria	Cauda equina syndrome	MRI: no abnormal	PMAI: Poor control of stool and urine	Yes but no compensation
21		No	Cesarean delivery	CSEA, L3-4	Uneventful puncture, epidural analgesia	Numbness in the lumbosacral and perineal region, urinary and fecal incontinence, loss of sensation below the knees in both legs, muscle strength rated at 0–1	cauda equina syndrome	MRI: no abnormal	PMAI: Weakness of both legs	No

*TURP* Transurethral resection of the prostate; *EA* Epidural anesthesia; *CSEA* Combined spinal and epidural anesthesia; *SA* Single spinal anesthesia; *PMII* Permanent neurological minor injury; *PMAI* Permanent neurological major injury.

## Discussion

Our study was conducted over a ten-year period in the Guangxi, encompassing 2,723,615 cases of neuraxial anesthesia across 243 hospitals. This represents the only investigation into neurological complications following neuraxial anesthesia conducted within a provincial region in China. Our results indicated that the overall incidence of neurological complications following neuraxial anesthesia was 0.44‰, aligning with the findings reported by Brull and others^17^. The incidence of severe neurological complications, including spinal injury, CES, spinal hematoma, anterior spinal artery syndrome, and conus medullaris injury, was found to be 0.077‰, consistent with the results of Moen and Irestedt^18^. The incidence of permanent neurological complications was 0.011‰. Despite the extremely low incidence of neurological complications following neuraxial anesthesia and the fact that most patients achieved complete recovery post-treatment, a subset of patients experienced permanent neurological damage. This outcome has significant ramifications for affected patients, their families, and attending anesthesiologists.

This survey indicated a progressive reduction in the incidence of neurological complications associated with neuraxial anesthesia in Guangxi over the decade spanning from 2013 to 2022. This decline may be attributed to several factors, including heightened awareness of neurological complications among anesthesiologists in Guangxi, the implementation of epidural wire catheters, enhancements in the types of local anesthetics utilized, and advancements in the techniques of epidural and spinal anesthesia^22–24^. Based on the survey results, it can be concluded that the incidence of neurological injury complications associated with EA was the lowest, suggesting that neuraxial anesthesia should prioritize the use of EA. Among patients undergoing SA and CSEA, 10.4% (110 out of 1,055 patients) had puncture points located at the L2-L3 intervertebral space. Existing literature indicates that anesthesiologists demonstrate an accuracy of only 29% in anatomically identifying intervertebral spaces^25^, which frequently exceeds the actual puncture site. This discrepancy may contribute to the higher incidence of neurological complications observed following SA and CSEA than following EA. Studies have demonstrated that intrathecal administration of ropivacaine exhibited the lowest neurotoxicity when compared to intrathecal injections of levobupivacaine, bupivacaine, procaine, and lidocaine^26,27^. Consequently, ropivacaine is recommended as the preferred local anesthetic for intrathecal injection in Guangxi.

A prospective study identified multiple attempts at epidural placement, paresthesia during insertion, history of neuraxial anesthesia, and use of patient-controlled epidural analgesia as risk factors for neurologic complications^28^. Within the surveyed population, 6.6% of the patients with neurological complications experienced three or more attempts at neuraxial needle insertion. The success rate of single punctures in neuraxial anesthesia was 82.5%. However, the records of puncture attempts for 347 patients were absent, which may have contributed to a decreased likelihood of successful single punctures being documented. Therefore, for patients in unique clinical scenarios, such as those with edema or at an elevated risk of neurological complications due to challenging puncture sites, it is recommended to utilize ultrasound guidance whenever feasible^29^. Additionally, SA or CSEA should ideally be administered at the L3-L4 interspace.

Among the 1160 patients experiencing neurological complications, 48.8% utilized patient-controlled epidural analgesia. The existing literature indicates that the continuous administration of local anesthetics into the nerve or nerve bundles, particularly following the misplacement of the epidural catheter into the intervertebral foramen, can result in altered nerve function and blockade^30^; therefore, if patients exhibit nerve root stimulation during the puncture process, it is not recommended to use an epidural analgesia pump, and the depth of the epidural catheter should be limited to less than 6 cm^31^. Additionally, if patients experience sensations akin to electric shocks during the puncture process, the procedure should be halted, or the interspace should be adjusted.

Imaging findings of patients with neurological complications revealed varying degrees of lumbar disc herniation or spinal canal stenosis in this investigation. A study utilizing MRI examination showed anatomical abnormalities of the spinal canal in individuals with lumbar disc herniation, which may contribute to neurologic deficits resulting from neuraxial blockade^32^. Furthermore, patients presenting with preoperative lumbar nerve lesions have an elevated risk of postoperative neurological damage^33–35^. Consequently, patients with pre-existing central nervous system disorders (including multiple sclerosis, post-polio syndrome, amyotrophic lateral sclerosis, spinal stenosis, and disc diseases), inflammatory diseases (such as Guillain-Barré syndrome and postoperative inflammatory changes), and peripheral nervous system diseases (both hereditary and acquired, such as diabetic polyneuropathy and chemotherapy-induced peripheral neuropathy) should meticulously evaluate the advantages and disadvantages before opting for neuraxial anesthesia.

Therefore, neuraxial anesthesia must encompass a comprehensive assessment of the patient’s clinical status and adherence to standardized procedures to minimize the incidence of neurological complications. Meticulous post-anesthetic monitoring is essential to facilitate the identification of potential mismanagement and to mitigate the risk of neurologic injury, thereby enhancing overall patient outcomes. Additionally, this study is subject to several limitations. Firstly, being a retrospective analysis, the data were sourced from historical medical records, which may suffer from incomplete documentation or the absence of crucial variables, thereby potentially compromising the accuracy of the findings. Secondly, the 10-year duration of the study may encompass significant advancements in medical technologies, shifts in diagnostic criteria, and changes in data recording practices, thereby introducing temporal heterogeneity that could affect the consistency and comparability of the data. Despite efforts to conduct the investigation as thoroughly as possible, the results should be interpreted with caution.

## Conclusion

Despite the relatively low incidence of neurological complications associated with neuraxial anesthesia, the potential severity of these complications warrants significant clinical attention. In the context of perioperative management, it is imperative for healthcare professionals to maintain heightened vigilance in preventing and promptly detecting neurological complications.

## Data Availability

The data used or analyzed during the current study are available from the corresponding author on reasonable request.
